# Motivators and Barriers to Career Choices in Community Medicine Among Medical Students in South Punjab, Pakistan: A Cross-Sectional Survey

**DOI:** 10.7759/cureus.67991

**Published:** 2024-08-28

**Authors:** Raamish A Khan, Muneera Tariq, Ifra Sultan, Allahdad Khan, Abdul Ahad Riaz, Humna Shahzad, Muhammad Uzair, Ayesha Younus, Muhammad Zain Bin Shahid, FNU Poombal

**Affiliations:** 1 Community Medicine, Bakhtawar Amin Medical and Dental College, Multan, PAK; 2 Internal Medicine, Fatima Memorial Hospital College of Medicine and Dentistry, Lahore, PAK; 3 Community Medicine, Nishtar Medical University, Multan, PAK; 4 Public Health, Bakhtawar Amin Medical and Dental College, Multan, PAK; 5 Pathology, Nishtar Medical University, Multan, PAK

**Keywords:** south punjab, medical students, underserved regions, public health, curriculum development, preventive healthcare

## Abstract

Background

Community medicine plays a vital role in public health, yet research on medical students' career choices in this field is limited, especially in underserved areas like South Punjab, Pakistan. This study explores the factors that influence undergraduate medical students' interest in pursuing a career in community medicine.

Methodology

A cross-sectional survey was conducted with 305 fourth- and final-year undergraduate medical students from various colleges in South Punjab. Data were collected via a structured online questionnaire, focusing on students' interests, motivations, barriers, and perceptions related to community medicine.

Results

The survey found that 40% of students showed interest in community medicine, with higher interest among females. Key motivators included preventive healthcare and public health initiatives, while financial constraints and unclear career progression were significant barriers. A gap was noted between students' interest and their perceived preparedness to address public health challenges.

Conclusion

Female students showed greater interest in community medicine and were more likely to recommend it. Bridging the gap between interest and perceived preparedness requires enhancing practical experiences, increasing the visibility of community medicine successes, and addressing financial and career progression concerns. Implementing these strategies can help attract and retain students in community medicine and improve public health outcomes.

## Introduction

Community medicine is essential for public health, focusing on primary healthcare to improve population health outcomes. It equips medical students with the skills necessary to address public health challenges through evidence-based practices, teamwork, and ethical conduct [1]. By bridging individual and community health, community medicine emphasizes the management of disease prevention and health promotion at the community level [2].

Pakistan’s healthcare system encounters significant challenges, including uneven distribution of resources and varying public health needs. Community medicine doctors play a vital role in delivering a wide range of services, from preventive care to the management of acute and chronic conditions [3]. On a global scale, the health landscape is characterized by a "triple burden" of diseases, which includes persistent infectious diseases such as tuberculosis, HIV/AIDS, and malaria; the emergence and re-emergence of new pathogens like COVID-19 and the resurgence of diseases like measles; and a notable increase in chronic non-communicable diseases (NCDs) such as diabetes, heart disease, and cancer [4]. This triple burden reflects the complexities introduced by globalization, urbanization, and technological advancements, which, while improving health outcomes in some areas, also contribute to new health risks like sedentary lifestyles and unhealthy diets [5]. In this context, community medicine doctors are crucial as they navigate these multifaceted health challenges by providing comprehensive care and addressing both old and emerging health threats at the local level.

In contrast, South Punjab, Pakistan, faces what is known as a "double burden of disease," which specifically refers to the coexistence of two major health challenges in the region. The first challenge is a high prevalence of infectious diseases, such as tuberculosis and hepatitis, largely due to inadequate sanitation, limited access to clean water, and insufficient public health infrastructure. The second challenge is the rising incidence of chronic non-communicable diseases like diabetes and hypertension [6]. These conditions are becoming more common in South Punjab due to factors such as inadequate healthcare resources, low health literacy, and limited awareness about healthy lifestyle choices. Additionally, higher maternal and child mortality rates further complicate the healthcare landscape in the region [7,8]. Community medicine practitioners in South Punjab are crucial for addressing these health challenges. They implement preventive measures, provide health education, and manage diseases directly within the community. Their work includes improving health literacy, conducting disease surveillance, and contributing to policy development [9].

Despite the vital role of community medicine, there is limited research on medical students' career choices in this field, particularly in South Punjab. This study aims to explore these career choices and the factors influencing them, filling a significant gap in the existing literature [10,11]. By investigating students' perceptions, the study seeks to guide educational and policy strategies to attract more students to community medicine, ultimately enhancing healthcare delivery in the region [12]. The report will describe the methodology, present key findings about student interests, discuss the implications for educational practices and policy, and provide recommendations for curriculum improvements and recruitment strategies. Additionally, it will suggest future research directions to further explore community-oriented medical education and its impact on healthcare outcomes.

## Materials and methods

Study design

This study was designed as a cross-sectional survey to understand the attitudes of fourth- and final-year undergraduate medical students in South Punjab towards community medicine as a career option.

Study setting and population

The survey was conducted among fourth- and final-year undergraduate medical students enrolled at various medical colleges in South Punjab, including Nishtar Medical University, Bakhtawar Amin Medical and Dental College, and Multan Medical and Dental College.

Sample size and sampling technique

A total of 305 students participated in the study. Participants were selected using a convenience sampling technique, involving inviting all fourth- and final-year undergraduate medical students from the specified colleges to participate in the survey.

Inclusion criteria

Fourth- and final-year undergraduate medical students enrolled at the specified colleges. Students who provided informed consent to participate in the survey.

Exclusion criteria

Students who were not in their fourth or final year of undergraduate medical school. Students who were on academic leave or had suspended their studies during the survey period. Students who had already chosen a specialty or career path other than community medicine. Students with incomplete or inconsistent survey responses that could not be used for analysis.

Sample size calculation

The sample size was calculated using the formula for estimating proportions, considering a 95% confidence level and a 5% margin of error. Based on an estimated population of 1,200 fourth- and final-year undergraduate students across the colleges, the calculated sample size was approximately 290. A total of 305 students participated.

Data collection

Data were collected through an anonymous online survey. A structured questionnaire was developed to assess various factors influencing students' attitudes towards community medicine. The questionnaire included sections on demographic information (age, gender, year of study, college affiliation), interest levels in community medicine, appealing aspects of the field, the likelihood of recommending community medicine to peers, and factors influencing perceptions (such as past experiences and relevance of topics covered in their education). Additionally, it included questions about perceived obstacles to choosing community medicine and suggestions for improving its image.

Quality assurance

The questionnaire was pre-tested on a small group of students to ensure clarity and relevance. Feedback from this pre-test was used to refine the questionnaire. The final version was reviewed by experts to ensure content validity.

Data collection procedures

To ensure one-time participation, the online survey employed the Completely Automated Public Turing Test to Tell Computers and Humans Apart (CAPTCHA) and monitored Internet Protocol (IP) addresses. Students received invitations via email and institutional announcements, with reminders sent to increase response rates.

Data analysis

Data analysis was performed using IBM SPSS Statistics for Windows, Version 25 (Released 2017; IBM Corp., Armonk, New York, United States). Descriptive statistics summarized the demographic characteristics of the participants. The normality of the data was assessed using the Shapiro-Wilk test and the Kolmogorov-Smirnov test. Both tests resulted in very small p-values, indicating that the data does not follow a normal distribution. Additionally, visual inspections through a histogram and Q-Q plot confirmed the non-normal distribution. Therefore, non-parametric statistical tests were used for further analysis. Chi-square tests were used to explore associations between demographic variables and students' attitudes towards community medicine, with a significance level set at p < 0.05.

Ethical considerations

The study was conducted online and did not involve collecting identifiable personal information. However, ethical clearance, referenced as IRB/0252/23, was obtained from the Institutional Review Board (IRB) of Bakhtawar Amin Medical and Dental College to confirm adherence to ethical research practices.

## Results

Demographic information

**Figure 1:**
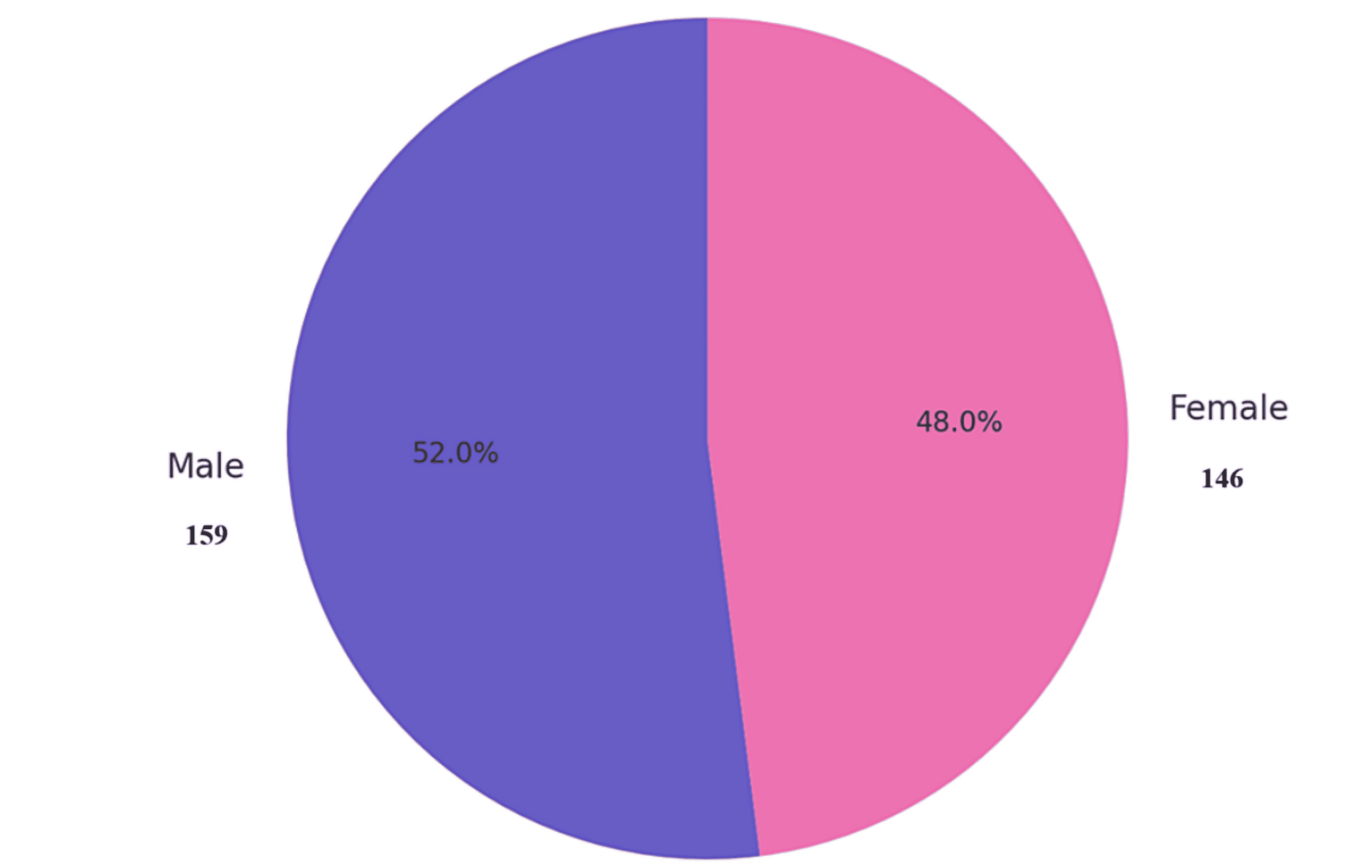
Gender distribution (n=305)

**Figure 2:**
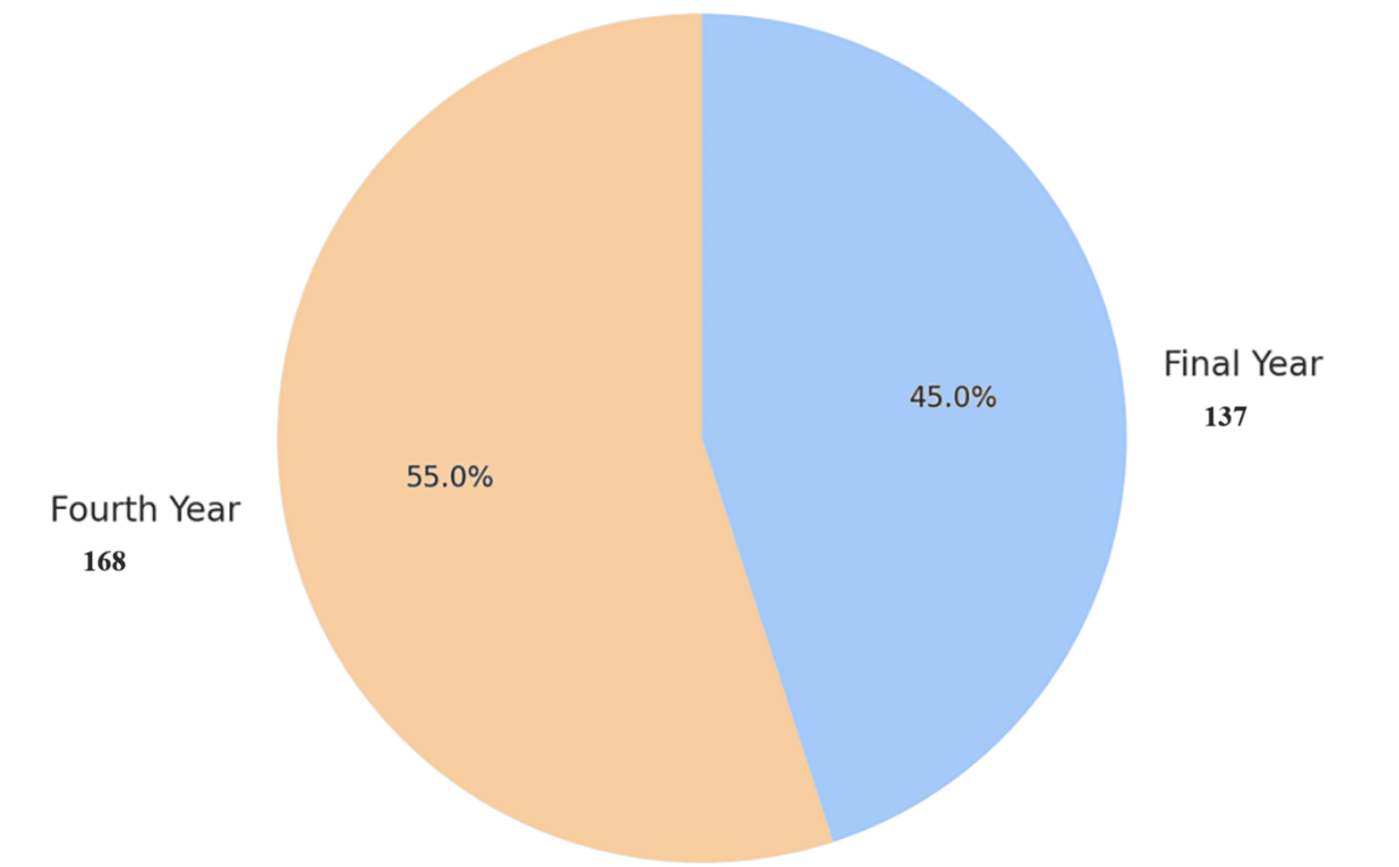
Year of study distribution of participants (n=305)

A total of 305 medical students participated in the study, comprising 159 (52%) males and 146 (48%) females. The distribution across academic years was 168 (55%) in the fourth year and 137 (45%) in the final year (Figures 1, 2). Students were from various colleges, with the highest representation from Nishtar Medical University (93, 30.5%) and Quaid-e-Azam Medical College (58, 19.0%). Bakhtawar Amin Medical and Dental College contributed 52 students (17.1%) (Table 1).

**Table 1:** College distribution of the participants (n=305)

College	n (percentage)
Bakhtawar Amin Medical And Dental College (BAMDC)	52 (17.1%)
Quaid-e-Azam Medical College (QAMC)	58 (19%)
Nishtar Medical University (NMU)	93 (30.5%)
Combined Military Hospital (CMH) Multan	60 (19.7%)
Multan Medical and Dental College (MMDC)	14 (4.6%)
D.G Khan Medical College	6 (2%)
Others (Sheikh Zayed Medical College Rahim Yar Khan, Shahida Islam Medical College, Combined Military Hospital Bahawalpur)	22 (7.1%)

Attitude towards community medicine as a career option

**Figure 3:**
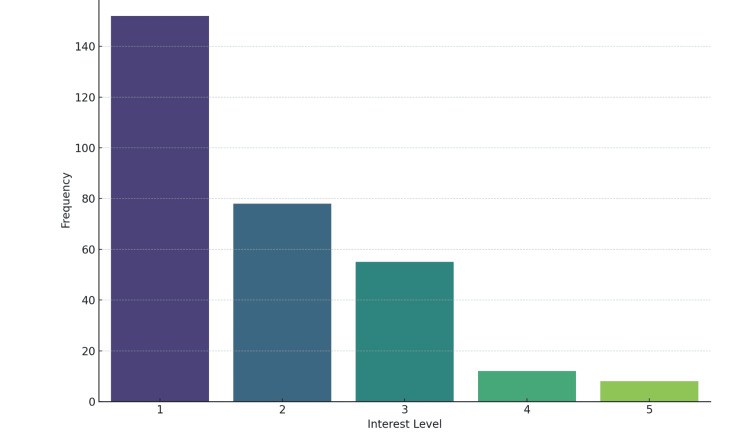
Interest in pursuing a career in community medicine (n=305)

**Figure 4:**
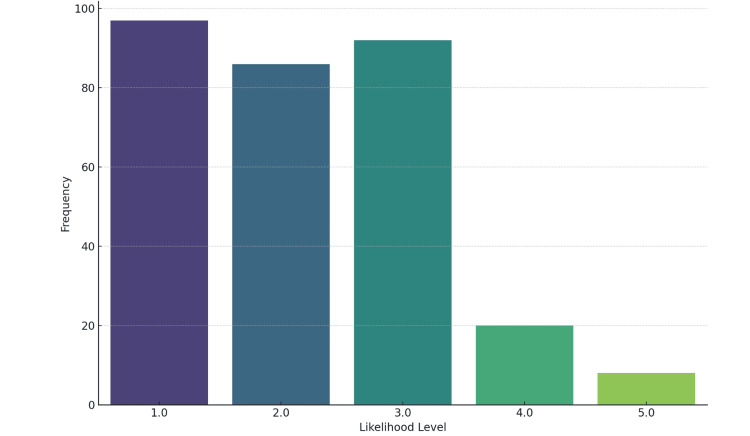
Likelihood of recommending community medicine to their peers (n=305)

**Figure 5:**
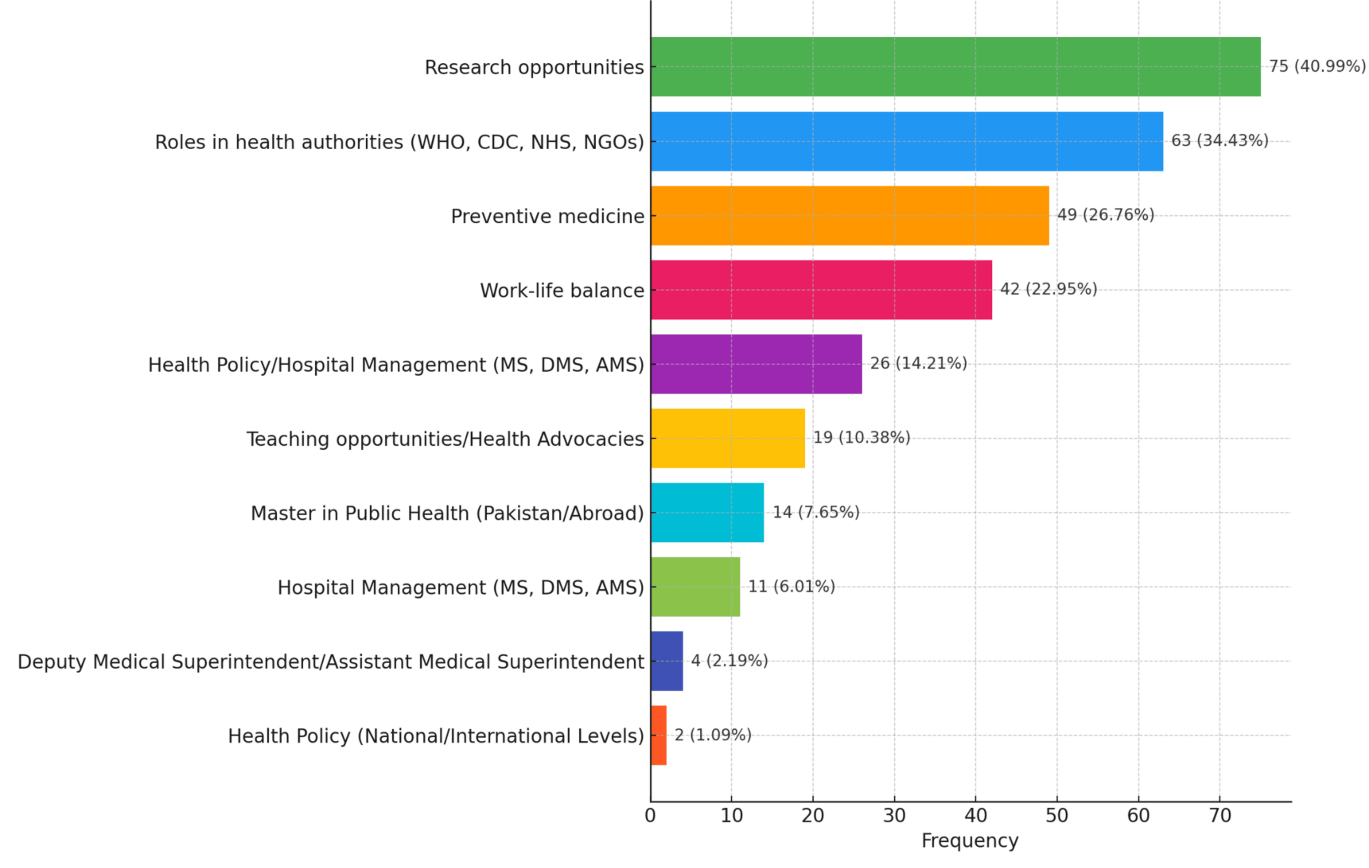
Interest in the aspects of community medicine (n=305)

A moderate level of interest in community medicine as a career choice was observed, with 122 (40%) students expressing interest (scoring four or five on a five-point scale) (Figure 3). Among those interested, the most appealing aspects were preventive healthcare (79, 25.90%), infectious disease control (61, 20%), and public health campaigns (56, 18.36%). However, only 107 (35%) students felt comfortable recommending community medicine to peers (rated four or five on a five-point scale) (Figure 4). Only 92 (30%) felt fully prepared to address public health challenges in South Punjab (rated four or five on a five-point scale) (Figure 5).

Factors influencing perception

Medical school curricula significantly shaped student perceptions of community medicine, with 238 (78.22%) indicating that field visits and coursework positively influenced their attitudes. The most relevant topics to students were public health campaigns (56, 18.36%), preventive healthcare initiatives (79, 25.90%), and infectious disease control (61, 20%). Major obstacles included a lack of clear career progression (104, 34.10%), insufficient exposure during medical school (70, 22.95%), and perceived lower financial rewards (54, 17.70%) (Table 2).

**Table 2:** Analysis of community medicine attitudes (n=305)

Factor	Option	Frequency	Percentage	p-value
Preparedness to address public health challenges	Very unprepared (1)	71	23.28%	0.04
	2	81	26.56%	-
	3	116	38.03%	-
	4	30	9.84%	-
	Very prepared (5)	07	2.30%	-
Interactions with local communities shaping views	Yes	221	72.46%	0.02
	No	84	27.54%	-
Most relevant aspects of community medicine	Public health campaigns	56	18.36%	0.02
	Preventive healthcare initiatives	79	25.90%	-
	Infectious disease control	61	20%	-
	Family planning and maternal health	74	24.26%	-
	Immunization	28	9.18%	-
	Occupational health	07	2.30%	-
Main obstacles or concerns	Perceived lower financial rewards	54	17.70%	0.01
	Insufficient exposure during medical education	70	22.95%	-
	Limited specialization opportunities	65	21.31%	-
	Lack of clear career progression	104	34.10%	-
	Heavy workload and time commitments	12	3.93%	-

Strategies to improve community medicine's image

Several strategies emerged as potential solutions to improve student perceptions of community medicine careers. The most preferred strategies were more hands-on experiences in the curriculum (30.49%), mentorship programs with community health specialists (25.25%), and increased visibility of community medicine achievements (21.64%) (Table 3). Additionally, addressing mental health aspects within community medicine (31.48%) and incorporating the role of technology (20.33%) were also highlighted as important. Guest lectures by experienced practitioners (11.15%) and incentives for choosing community medicine (11.48%) were also considered valuable.

**Table 3:** Analysis of improvement in the perception and additional aspects of community medicine (n=305)

Factor	Option	Frequency	Percentage	p-value
Improving the perception of community medicine	Guest lectures by experienced practitioners	34	11.15%	0.03
	Increased visibility of community medicine achievements	66	21.64%	-
	Mentorship programs with community health specialists	77	25.25%	-
	More hands-on experiences in the curriculum	93	30.49%	-
	Incentives for choosing community medicine	35	11.48%	-
Additional aspects to be addressed	The role of technology in enhancing community medicine practices	62	20.33%	0.04
	Addressing mental health aspects within community medicine	96	31.48%	-
	Cultural considerations influencing community healthcare practices	58	19.02%	-
	Strategies for improving collaboration between community medicine and other medical specialties	83	27.21%	-
	Other	06	1.97%	-

Chi-square analyses revealed significant gender differences in several areas related to community medicine. There was a notable difference in interest in pursuing a career in community medicine by gender (χ² = 4.25, p = 0.03). Similarly, gender differences were observed in the likelihood of recommending community medicine as a career to peers (χ² = 3.89, p = 0.04). Additionally, preparedness to address public health challenges showed a significant association with gender (χ² = 4.10, p = 0.04) (Table 4). These results underscore the need for gender-sensitive approaches to enhance interest, advocacy, and preparedness in community medicine.

**Table 4:** Chi-square test results for gender and career interest in community medicine (n=305)

Test Name	Chi-Square (χ²)	p-value
Interest in career vs. gender	4.25	0.03
Recommend to peers vs. gender	3.89	0.04
Preparedness vs. public health challenges	4.10	0.04

## Discussion

Our study offers insightful perspectives on medical students' career preferences in community medicine, revealing significant trends and areas for improvement. The results indicate notable gender differences in interest and preparedness, aligning with broader trends observed in medical education [13]. 

The findings show that female medical students in South Punjab exhibit a stronger inclination towards community medicine compared to their male counterparts [14]. This preference aligns with previous studies suggesting that female students often favor public health and community-oriented roles. The increased interest among female students may be attributed to their inclination toward preventive care, public health initiatives, and research, which are central to community medicine [15]. This pattern reflects a broader trend where female medical students prioritize holistic and community-based health approaches [16].

The lack of a significant difference in preparedness between fourth-year and final-year students highlights the need for a more integrated approach to community medicine education throughout medical school [17]. To better prepare students for careers in community medicine, educational institutions should enhance curricula by incorporating more practical experiences and field visits. Integrating hands-on training, community projects, and real-world case studies into the coursework can provide students with a deeper understanding of the field [18]. Additionally, innovative teaching approaches, such as the use of simulations, are crucial for equipping public health practitioners with the skills needed to handle diverse health scenarios, as these methods allow students to engage in immersive learning experiences that closely mimic real-world conditions [19].

Our study also identified several challenges that impact students' perceptions of community medicine. Perceived lower financial rewards, insufficient exposure during medical education, and limited career pathways were major obstacles. Addressing these concerns through clear information on potential salaries, career advancement opportunities, and success stories can make the field more attractive to students [20]. Additionally, integrating mentorship programs, collaborations with local health organizations, and guest lectures from experienced practitioners can further enrich students' experiences and inspire them to pursue careers in community medicine [21,22].

One limitation of our study is the potential response bias, as students with a pre-existing interest in community medicine may have been more inclined to participate, potentially skewing the results [23]. Additionally, the study's focus on a specific geographic location limits the generalizability of findings to other low-income regions. Despite these limitations, our study provides valuable insights for future research and educational strategies.

Community medicine is increasingly recognized as a critical medical stream, with a growing emphasis on public health and preventive care. The field's focus on addressing community health needs and improving public health outcomes aligns with global trends and the evolving healthcare landscape [24].

Looking ahead, future directions for community medicine include expanding simulation-based training and enhancing practical experiences for public health practitioners. Simulation and hands-on training are essential for bridging the gap between theoretical knowledge and practical application. Implementing these advancements will improve the effectiveness of community medicine education and better prepare students for real-world challenges in the field [1,25].

## Conclusions

This study explored medical students' perceptions of community medicine careers in South Punjab, Pakistan. It found that female students expressed a greater interest in and were more likely to recommend community medicine compared to their male peers. The results highlight the need for enhanced educational strategies to address concerns about financial rewards and career progression. Incorporating practical experiences, such as field visits and mentorship opportunities, is crucial for preparing future community medicine practitioners effectively. These improvements could help attract and retain more students in this vital field, ultimately benefiting community health.
